# Screening Early Renal Disease Using Albuminuria in Indian Patients at High Risk

**DOI:** 10.1016/j.ekir.2026.106605

**Published:** 2026-05-14

**Authors:** Narayan Prasad, Sanjeev Gulati, Anil Kumar Bhalla, Arpita Raychaudhuri, Manisha Sahay, Santosh Varughese, Bharath HS, Ranjini Sen, Shyam Bihari Bansal

**Affiliations:** 1 Department of Nephrology, Sanjay Gandhi Postgraduate Institute of Medical Sciences, Lucknow, India; 2 Department of Nephrology and Kidney Transplant, Fortis Group of Hospitals, National Capital Region, India; 3 Department of Nephrology, Sir Ganga Ram Hospital, New Delhi, India; 4 Department of Nephrology, IPGMER and SSKM Hospital, Kolkata, India; 5 Department of Nephrology, Osmania General Hospital, Hyderabad, Telangana, India; 6 Department of Nephrology, Christian Medical College, Vellore, Tamil Nadu, India; 7 Medical Affairs, AstraZeneca Pharma India Ltd, Bengaluru, Karnataka, India; 8 Department of Nephrology, Medanta – The Medicity, Gurugram, Haryana, India

**Keywords:** albuminuria, chronic kidney disease, diabetes mellitus, hypertension, India

## Introduction

Chronic kidney disease (CKD) is characterized by gradual loss of kidney function, based on low estimated glomerular filtration rate (eGFR < 60 ml/min per 1·73 m^2^), elevated albumin-creatinine-ratio (ACR > 30 mg/g), or both for ≥ 3 months.^1^^,^^2^ CKD can be asymptomatic in early-stages and gradually progress to end-stage kidney disease, requiring dialysis or kidney transplant, affecting 1 in 10 people worldwide, with its burden rising over past 2 decades.^3^ It is projected to be the fifth most common cause of years-of-life lost globally by 2040.^4^^,^^5^ No decline has been observed in the global age-standardized mortality rate. Early CKD-identification is crucial for delaying end-stage kidney disease progression and reducing its economic burden.^3^^,^^4^^,^^6^ CKD is a major risk-factor for cardiovascular deaths and for preventable and treatable complications, including diabetes and hypertension.^6^

India contributes to the global CKD-burden, with an estimated 140.2 million cases,^7^ owing to its large demographics and exceptionally high prevalence of diabetes, hypertension, and obesity.^6^^,^^8^^,^^9^ According to the Indian Council of Medical Research-(ICMR)-INDIAB national cross-sectional study, 11.4%, 35.5%, and 28.6% of Indians have diabetes, hypertension, and generalized obesity, respectively.^9^ The coexistence of these diseases causes a complex interplay, leading to diabetes-hypertension-kidney-disease-(DHKD) syndrome, highlighting an “epidemic” condition in India.S1,S2 Also, cardiovascular kidney metabolic syndrome can result in multisystem morbidity, attributed to the interrelationship between obesity, diabetes, CKD, and cardiovascular diseases.S3 End-stage kidney disease (ESKD) management is resource-intensive, and limited access to dialysis and kidney transplants in India worsens CKD-related morbidity and mortality. The Million Death Study 2017 reported an increase by 38% in kidney failure-related deaths in India from 2001–2003 to 2010–2013,S4 and found significant association of increased kidney failure deaths with diabetes, hypertension, and cardiovascular disease, with diabetes being strongest predictor (odds ratio vs. control [95% confidence interval]:15·1, [12·6–18·1] in 2010–2013).S4 CKD represents a major burden in low-middle-income-countries like India, where the government’s focus is on basic healthcare facilities for rural areas.S5

Population-level screening to identify high-risk populations, with increased awareness among primary care physicians, is a necessary preventive measure to avoid CKD. Early CKD detection by targeted screening has shown cost-effectiveness and improvement in quality-adjusted life years.S6,S7 The International Society of Nephrology (ISN),S8 National Kidney Foundation,S9 Kidney Disease: Improving Global Outcomes (KDIGO),S10 and the Asian Forum for CKD InitiativesS11 recommend regular CKD-screening in at-risk individuals.

According to the National Kidney Foundation, proteinuria and eGFR are key factors in risk-stratification of patients with CKD.S11 The decrease in GFR provides information regarding CKD-stage and associated comorbidities.S12,S13 Similarly, albuminuria, commonly measured as spot urine albumin-to-creatinine ratio (UACR) is another key biomarker considered in the cross-classification table of KDIGO guidelines (Supplementary Figure S1),S14-S17 suggesting an association between CKD progression and albuminuria in normoalbuminuria range. The dipstick-based UACR testing is cost-effective, accessible, and feasible in routine clinical settings, and thus widely used for CKD-screening in low- and middle-income-countries.S18

Screening Early Renal Complication in High-Risk- (SEARCH) patients is a population-level diagnostic evaluation to screen for proteinuria using urine dipstick-based UACR testing in India (Table 1). This SEARCH evaluation aimed to educate primary care physicians on early CKD diagnosis and aid prompt management of at-risk patients. In this study, we estimated the proportion of patients with abnormal UACR among high-risk patients with diabetes and/or hypertension.

**Table 1:** UACR test results based on the spot dipstick urinalysis

Creatinine levels	Microalbumin (mg/l)		
	< 0.08 g/l	0.08 g/l	0.12 g/l
Creatinine (mmol/l)	UACR (mg/mmol)		
0.9	Normal (< 0.08)	88.89	133.33
4.4		18.18	27.27
8.8		9.09	13.64
17.6		4.55	6.82
26.5		3.02	4.53

UACR, urinary albumin-to-creatinine ratio.

Abnormal UACR values (>3.04 mg/mmol).

## Results

Between March 10, 2022 and April 25, 2022, a total of 44,037 patients were screened across India. The mean (SD) age of the patients was 53.5 (13.1) years, and 57% were men. Most patients had both diabetes and hypertension (42%), followed by diabetes only (31%) and hypertension only (14%; Supplementary Table S1).

### UACR Test Findings

Of the 43,109 evaluable patient data, 35.6% (*n* = 15,334) of patients had an abnormal UACR (> 30 mg/g). The mean (SD) age was 56.9 (12.7) years, and 60% were men. The number (%) of patients with moderately increased albuminuria (UACR = 30–299 mg/g) was 11,776 (27%), whereas 3233 (8%) of patients had severely increased albuminuria (UACR > 300 mg/g). The proportion of patients with abnormal UACR was higher (50.1%; 9353/18,668) among patients with both diabetes and hypertension, whereas among patients with diabetes only and hypertension only, proportion of patients with abnormal UACR was 27.0% (3686/13,673) and 22.8% (1427/6271), respectively (Supplementary Table S1).

### eGFR Staging in At-Risk Population

The eGFR data were available for 1268 patients. Most (*n* = 947; 75%) had eGFR > 60 ml/min; among these, 30% to 49% of patients had abnormal UACR.

**Figure 1:**
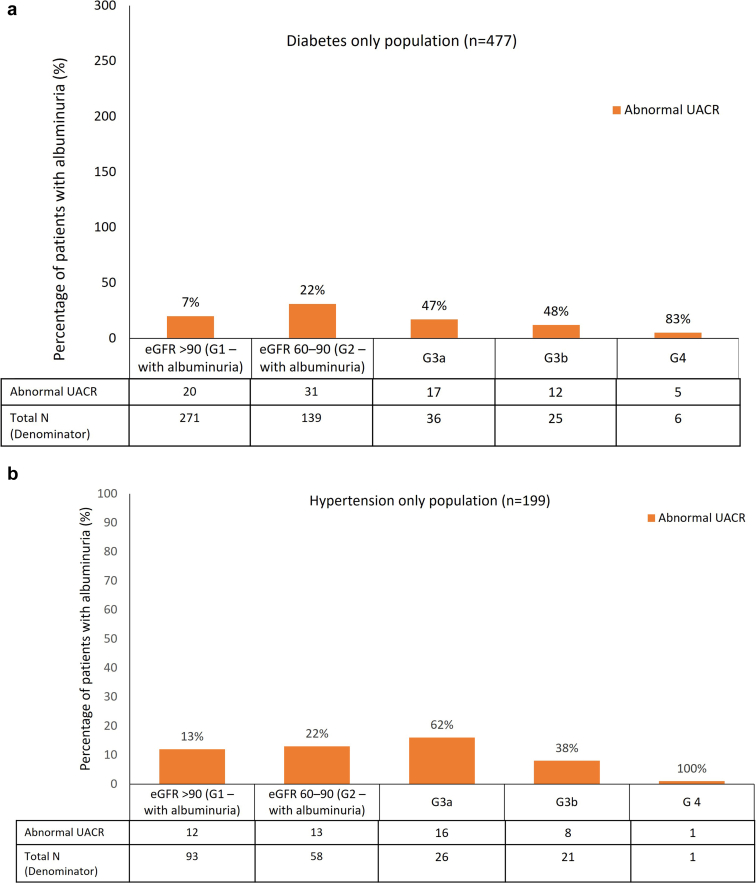

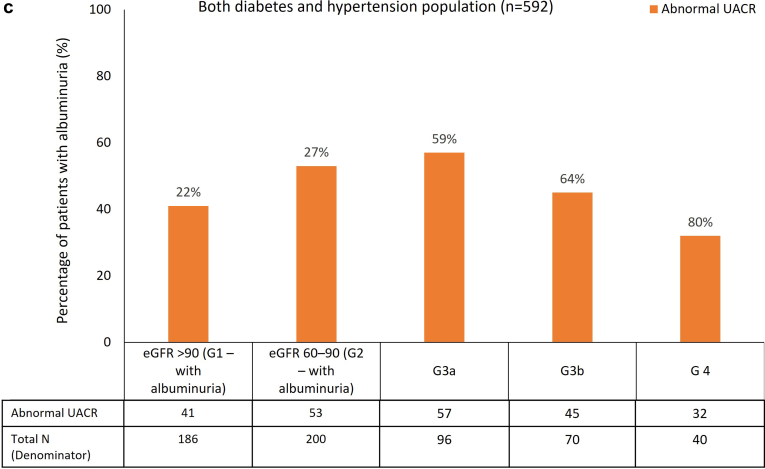
Distribution of eGFR categories and albuminuria prevalence among patients with (a) diabetes, (b) hypertension, and (c) both diabetes and hypertension population. (a) Diabetes only population (*n* = 477). (b) Hypertension only population (*n* = 199). (c) Both diabetes and hypertension population (*n* = 592). *N* = total number of patients; *n* = patients with abnormal UACR. CKD stages G1 and G2 are defined only in the presence of albuminuria (UACR ≥ 30 mg/g), in accordance with KDIGO guidelines. CKD, chronic kidney disease; eGFR, estimated glomerular filtration rate; UACR, urine albumin-creatine ratio.

The prevalence of albuminuria (UACR ≥ 30 mg/g) increased with worsening kidney function across all groups (Figure 1a–c). Among patients with diabetes, albuminuria was observed in 7% of individuals with eGFR > 90 ml/min/1.73 m^2^ and 22% with eGFR 60 to 89 ml/min/1.73 m^2^, with higher proportions in CKD G3a to G4 (Figure 1a). A similar trend was seen in hypertension, with corresponding prevalences of 13% and 22% (Figure 1b). Patients with both diabetes and hypertension had the highest burden, with albuminuria present in 22% and 27% of individuals with eGFR > 90 and 60 to 89 ml/min per 1.73 m^2^ respectively, increasing further in CKD G3a to G4 (Figure 1c). Individuals with eGFR ≥ 60 ml/min per 1.73 m^2^ were not classified as having CKD unless albuminuria was present; accordingly, CKD G1 and G2 included only those with UACR ≥ 30 mg/g, consistent with KDIGO definitions.

## Discussion

The SEARCH was a pan-India CKD diagnostic evaluation involving the screening of > 43,000 participants to determine abnormal UACR prevalence in high-risk patients with diabetes and hypertension/both. We reported that over one-third (> 35%) of the total population showed abnormal UACR > 30 mg/g, suggesting that hypertension and diabetes are important CKD predictors or risk-factors.^7^^,^S19 We identified abnormal UACR in the younger population (< 55 years) with equal gender distribution, contrasting global CKD trends having higher-prevalence in older adults (> 60 years) with historical male predominance.S20 This suggests that CKD risk-factors may affect Indian patients earlier than seen in global studies, highlighting the need for early-screening in high-risk populations.

This study reported most patients with diabetes (31%) and lower proportion with hypertension (14%) compared with the ICMR-INDIAB national study (11.4% and 35.5%, respectively).^9^ This difference is likely because of our healthcare-facility-based recruitment, which captured more patients actively managing diabetes, whereas many with hypertension remain undiagnosed or asymptomatic and less likely to seek routine care. Diabetes was the most common comorbid condition; nearly half of the population had both diabetes and hypertension. Approximately 75% had CKD stage 1 or 2 with eGFR ≥ 60 ml/min per 1.73 m^2^, indicating preserved kidney function; among these, a substantial proportion had albuminuria. Population-based data on CKD-prevalence in India are scarce. However, state- or region-level data are available.S21–S26 Our findings align with previous research showing high-burden of albuminuria in India (Supplementary Material).

The strengths of our study include screening of a large-sample-size with broader regional coverage, use of standardized urine dipstick coloring grade to measure UACR, and alignment with KDIGO guidelines (for use of urine dipstick-testing for proteinuria). Our findings provide latest data on proteinuria prevalence across India, adding valuable information on CKD risk-factors. The analysis shows high abnormal UACR burden among high-risk patients with diabetes and hypertension in healthcare settings, possibly because of rising UACR-prevalence than in general population. Future community-based studies are needed to assess true prevalence.

This assessment is limited in its ability to impact generalizability of findings. Our convenient sampling design may limit accurate evaluation of population-level-prevalence. We could not adjust relevant confounding factors, including disease duration, treatments, and lifestyle-related-factors associated with CKD-risk. We reported descriptive data, and robust statistical evaluation of prevalence would be of additional value. One-time UACR-measurement may have overestimated proteinuria prevalence. CKD-staging lacked uniform eGFR-estimation equation, and was based on limited data, underrepresenting India’s true disease burden.

In conclusion, our diagnostic analysis findings raise concern regarding high-burden of proteinuria, an early sign of CKD, among patients with diabetes and hypertension. Screening warrants early intervention to improve CKD outcomes in at-risk patients. The urine UACR-dipstick test is an effective population-screening method, and awareness among healthcare practitioners and patients is paramount in early diagnosis of CKD in at-risk patients. Single-dipstick urinalysis in at-risk patients could be cost-effective in the long run. These findings showcase the urgent need for planning population-level preventive health policies and resources for CKD management in India.

## Disclosure

BHS and RS are employees of AstraZeneca Pharma India Ltd. The other authors declared no competing interests.

## Patient Consent

The authors declare that this study was done in collaboration with ISN and data belongs to ISN.

## Supplementary Material

**Supplementary Methods**.

**Supplementary Discussion**.

**Supplementary Reference**.

**Figure S1**. Cross-classification of GFR and albuminuria as per KDIGO guidelines.

**Figure S2**. Project SEARCH: Participating states.

**Figure S3**. Colour chart on the dipstick bottle to estimate UACR.

**Figure S4**. Patient record form.

**Table S1**. Demographics, medical history, and clinical characteristics of participants in the diagnostic assessment.

**Table S2**. Early diagnosis of patients with CKD based on abnormal UACR.

**STROBE Checklist**.
