# Supplementary material for: Screening Early Renal Disease Using Albuminuria in Indian Patients at High Risk
Source: Kidney Int Rep. 2026 May 14;11(8):106605. doi: 10.1016/j.ekir.2026.106605 (PMC13314571; doi:10.1016/j.ekir.2026.106605)
Supplement: Supplementary file (PDF) [file mmc1.pdf]

Supplementary Materials

Supplementary Methods

Diagnostic Exploration Design and Population

The SEARCH was an observational, multicenter, cross-sectional, investigational screening conducted in the physicians' clinics or public sector hospitals across India (Figure 1). This population-level screening was carried out by the Indian Society of Nephrology (ISN), as part of the World Kidney Day 2022 awareness initiative between March 2022 to April 2022. Invitations to participate in this diagnostic screening were sent out by the survey sponsor and ISN to all participating centers. The screening program was approved by the governing body of ISN. After confirming their participation, over a period of four weeks consecutively, patients who came to the clinics/hospitals for their routine treatment were screened for eligibility for inclusion in the evaluation program. High-risk patients from healthcare facilities, aged ≥18 years of either gender with either type-2 diabetes both newly diagnosed (plasma fasting glucose ≥126 mg/dL or glycated hemoglobin [HbA1c] ≥6·5%), or hypertension (systolic blood pressure ≥140 mm Hg or diastolic blood pressure ≥90 mm Hg) or already on treatment were included in the diagnostic assessment. Patients with pre-existing CKD were excluded. The screening procedure was explained to the patients, and self-consent was obtained before participating in the study. Patient education booklets, CKD awareness posters, urine dipstick bottle packs (Urine Test Strips, AVE Science & Technology Co. Ltd., Hunan, China), and patient record forms to capture screening results were provided to each participating clinic/center. Institutional ethics board approvals were not required for this analysis. No patient personal identification data was used for the purpose of this publication. Patient confidentiality was ensured with end-to-end encryption of the data.

Data Collection and Screening Procedure

Patient age, gender, and history of diabetes or hypertension were recorded in the patient record form. Random, single-spot urine samples were obtained from the patients, and dipstick urinalysis was performed by the trained clinic assistants within 24 hours of sampling. Patients with conditions including urinary tract infection, congestive heart failure, uncontrolled hyperglycemia, uncontrolled hypertension, recent pyrexia, and pregnancy, which may interfere with urine albumin estimation, were excluded. Based on the color change on the dipstick, the presence of creatinine and albumin was quantified, and findings were recorded in the patient record form (Appendix). The completed patient record form was then sent electronically to the ISN centralized email for data curation and analysis.

Outcome Variables and Assessments

The color on the test strip corresponding to 0·9 mmol/l of creatine and <0·08 g/l of albumin was within the normal range (Table 1). The dip stick test had a sensitivity of 0.15 g/l to detect protein, 0.08 g/l to detect albumin levels, and 4.4 mmol/l to detect creatinine levels in urine. Spot UACR was computed in mg of microalbumin per g of creatinine (mg/g), with a UACR of <30 mg/g considered as normal or mildly increased. The albuminuria categories were defined according to KDIGO guidelines as: A1: <30 mg/g (normal to mildly increased), A2: 30–300 mg/g (moderately increased), A3: >300 mg/g (severely increased). Reporting of eGFR in patients with albuminuria was not mandatory as per the assessment plan. However, sites/physicians with concurrent blood creatinine data available for their patients voluntarily reported eGFR data. Therefore, eGFR was available only in a small number of patients.

- **Cross-classification of GFR and albuminuria**[S17]

Chronic kidney disease (CKD) staging was classified according to the KDIGO 2024 guidance, which combines eGFR categories with albuminuria severity, reflecting overall CKD risk by incorporating both kidney function and kidney damage (Supplementary Figure S1). This combined approach captures the independent and additive prognostic value of reduced GFR and increased albuminuria. GFR categories (mL/min/$1.73m^2$ or g/L) were defined as: Stage G1≥90 (normal) with albuminuria or other markers of kidney damage, Stage G2=60–89 (mildly decreased) with albuminuria or other markers of kidney damage, Stage G3a=45–59. Albuminuria categories were defined as A1 (< 30 mg/g), A2 (30–300 mg/g; corresponding to microalbuminuria), and A3 (> 300 mg/g). Prognostic risk increases progressively with declining GFR and increasing albuminuria, with the highest risk observed in patients with G4–G5 CKD and A3 albuminuria.

Data Analysis

There was no formal hypothesis testing done in this analysis, and hence, sample size and power calculation were not required. All data were summarized descriptively. The demographic data are summarized using mean, standard deviation (15) and range. The number (n [%]) of patients with abnormal UACR and eGFR based on CKD staging in the screened population, and among patients with diabetes, hypertension, or both, are reported. All statistical analyses were performed using SAS (version 9.4, USA).

Supplementary Results

The geographical distribution of the total Project SEARCH population was as follows: East region- 8%, West region- 20%, North region- 34%, South region- 38% (data not analyzed). Based on the abnormal-UACR among high-risk patients with diabetes, hypertension, and/or both, CKD-prevalence was

estimated as 8.37% (n=3686), 3.24% (n=1427) and 21.24% (n=9353), respectively, guiding physicians towards early CKD-diagnosis. (Supplementary Table S2)

Supplementary Discussion

A population-based study in Punjab reported 46.7% albuminuria prevalence, significantly higher than 8.9% reported in the United States.[S27] This substantial disparity underscores greater CKD burden in Indian populations. A study in Delhi and Chennai reported 8.7% CKD-prevalence (n=1/12), with 80% of affected undiagnosed individuals. Furthermore, diabetes (15%) and albuminuria (7.1%) prevalence support urgent need for screening in this population.[S21] The SEEK-India study, spanning broader region, reported 17.2% of CKD-prevalence, with ~80% having proteinuria.[S25] Another study in semi-urban-Western India also reported similar prevalence (18.6%), based on eGFR<60ml/min/1.73m^2 or proteinuria>1+ on dipstick.[S26] A study from Eastern India reported dipstick proteinuria≥1+ (7.7%), including participants with hypertension (~40%), and UACR (23.5%, 30–300mg/g).[S22] Among 12,500 individuals without known CKD risk-factors from urban-and rural-areas of Northern and Southern India, eGFR<60ml/min prevalence was 1.6%, and higher-prevalence (4.8%) was reported in rural-areas of Southern India.[S24] The Indian Chronic Kidney Disease-(ICKD) study reported albuminuria (using dipstick) and UACR>300mg/g in 41.4% and 25.5% of participants (N=4,056) with mild-to-moderate-CKD, respectively.[S21] Eastern Uttar Pradesh study reported India's one of the highest albuminuria prevalence (86.7%) among CKD patients and suggested association between chronic fluoride exposure in drinking water and increased risk of diabetic nephropathy leading to CKD[S28], highlighting role of environmental-risk-factors in CKD-prevalence. Despite methodological differences, these studies highlight high-CKD-prevalence in India, and align with our findings of high-prevalence of "silent-CKD" among Indian adults, underscoring need

for targeted screening and early intervention strategies. Interestingly, proteinuria screening is not regular and mandated investigation for diabetes and hypertension patients in India. Our findings support UACR-testing by dipstick method as a cost-effective tool for population-level proteinuria screening. Notably, albuminuria is crucial to determine risk associated with CKD and monitor progression in this population. Furthermore, patients with CKD and normoalbuminuria (<30mg/g) are also at-risk of linear progression.

Role of the Funding Source

The funder of the assessment contributed to the diagnostic screening design, data analysis, data interpretation, and writing of the report.

Supplementary References

S1. Dash SC, Agarwal SK, Panigrahi A, et al. Diabetes, Hypertension and Kidney Disease Combination "DHKD Syndrome" is common in India. *J Assoc Physicians India* 2018; 66: 30-33.
S2. Naveen Angadi AB, Raju H. Badiger, Abhiram Narasimha, Pavan Kumar B.C., Gayatri Sharma, Rishabh Agarwal, Sreekant N. Chowdary. Clinical and Laboratory Profile of Diabetic-Hypertension, Kidney Disease Syndrome - A Hospital Based Study in South India, https://www.sciencegate.app/document/10.18410/jebmh/2021/205 (2021, accessed 4 September 2023).

S3. Ndumele CE, Rangaswami J, Chow SL, et al.; American Heart Association. Cardiovascular-Kidney-Metabolic Health: A Presidential Advisory From the American Heart Association.Circulation. 2023;148(20):1606-1635.
S4. Dare AJ, Fu SH, Patra J, et al. Renal failure deaths and their risk factors in India 2001-13: nationally representative estimates from the Million Death Study. *Lancet Glob Health* 2017; 5: e89-e95. DOI: 10.1016/s2214-109x(16)30308-4.
S5. Foundation IBE. Healthcare industry in India, https://www.ibef.org/industry/healthcare-india (2023, accessed 4 September 2023).
S6. Yeo SC, Wang H, Ang YG, et al. Cost-effectiveness of screening for chronic kidney disease in the general adult population: a systematic review. *Clinical Kidney Journal* 2023. DOI: 10.1093/ckj/sfad137.
S7. Cusick MM, Tisdale RL, Chertow GM, et al. Population-Wide Screening for Chronic Kidney Disease : A Cost-Effectiveness Analysis. *Ann Intern Med* 2023; 176: 788-797. 20230523. DOI: 10.7326/m22-3228.
S8. Li PK-T, Weening JJ, Dirks J, et al. A report with consensus statements of the International Society of Nephrology 2004 Consensus Workshop on Prevention of Progression of Renal Disease, Hong Kong, June 29, 2004. *Kidney International* 2005; 67: S2-S7. DOI: 10.1111/j.1523-1755.2005.09401.x.
S9. Vassalotti JA, Stevens LA and Levey AS. Testing for Chronic Kidney Disease: A Position Statement From the National Kidney Foundation. *American Journal of Kidney Diseases* 2007; 50: 169-180. DOI: https://doi.org/10.1053/j.ajkd.2007.06.013.
S10. Levey AS, Atkins R, Coresh J, et al. Chronic kidney disease as a global public health problem: Approaches and initiatives – a position statement from Kidney Disease Improving Global Outcomes. *Kidney International* 2007; 72: 247-259. DOI: https://doi.org/10.1038/sj.ki.5002343.
S11. Li PK, Chow KM, Matsuo S, et al. Asian chronic kidney disease best practice recommendations: positional statements for early detection of chronic kidney disease from Asian Forum for Chronic Kidney Disease Initiatives (AFCKDI). *Nephrology (Carlton)* 2011; 16: 633-641. DOI: 10.1111/j.1440-1797.2011.01503.x.
S12. K/DOQI clinical practice guidelines for chronic kidney disease: evaluation, classification, and stratification. *Am J Kidney Dis* 2002; 39: S1-266.
S13. Current Chronic Kidney Disease (CKD) Nomenclature used by Kdigo. *Kidney International Supplements* 2013; 3: viii. DOI: https://doi.org/10.1016/S2157-1716(16)30005-3.
S14. Gerstein HC, Mann JF, Yi Q, et al. Albuminuria and risk of cardiovascular events, death, and heart failure in diabetic and nondiabetic individuals. *Jama* 2001; 286: 421-426. DOI: 10.1001/jama.286.4.421.
S15. Klausen K, Borch-Johnsen K, Feldt-Rasmussen B, et al. Very low levels of microalbuminuria are associated with increased risk of coronary heart disease and death independently of renal function, hypertension, and diabetes. *Circulation* 2004; 110: 32-35. 20040621. DOI: 10.1161/01.Cir.0000133312.96477.48.

S16. Gansevoort RT, Matsushita K, van der Velde M, et al. Lower estimated GFR and higher albuminuria are associated with adverse kidney outcomes. A collaborative meta-analysis of general and high-risk population cohorts. *Kidney Int* 2011; 80: 93-104. 20110202. DOI: 10.1038/ki.2010.531.
S17. Kidney Disease: Improving Global Outcomes (KDIGO) CKD Work Group. KDIGO 2024 Clinical Practice Guideline for the Evaluation and Management of Chronic Kidney Disease. Kidney Int. 2024;105(4S): S117–S314.

S18. Konta T, Hao Z, Takasaki S, et al. Clinical utility of trace proteinuria for microalbuminuria screening in the general population. *Clin Exp Nephrol* 2007; 11: 51-55. 20070328. DOI: 10.1007/s10157-006-0458-z.

S19. Talukdar R, Ajayan R, Gupta S, Biswas S, Parveen M, Sadhukhan D, et al. Chronic Kidney Disease Prevalence in India: A Systematic Review and Meta-Analysis From Community-Based Representative Evidence Between 2011 to 2023. *Nephrology* (Carlton). 2025;30(1):e14420.
S20. Li PK, Garcia-Garcia G, Lui SF, Andreoli S, Fung WW, Hradsky A, et al. Kidney health for everyone everywhere-from prevention to detection and equitable access to care. *Kidney Int.* 2020;97(2):226-32.
S21. Anand S, Shivashankar R, Ali MK, Kondal D, Binukumar B, Montez-Rath ME, et al. Prevalence of chronic kidney disease in two major Indian cities and projections for associated cardiovascular disease. *Kidney Int.* 2015;88(1):178-85.
S22. Gallieni M, Aiello A, Tucci B, Sala V, Brahmochary Mandal SK, Doneda A, et al. The burden of hypertension and kidney disease in Northeast India: the Institute for Indian Mother and Child noncommunicable diseases project. *ScientificWorldJournal.* 2014;2014:320869.
S23. Kumar V, Yadav AK, Sethi J, Ghosh A, Sahay M, Prasad N, et al. The Indian Chronic Kidney Disease (ICKD) study: baseline characteristics. *Clin Kidney J.* 2022;15(1):60-9.
S24. O'Callaghan-Gordo C, Shivashankar R, Anand S, Ghosh S, Glaser J, Gupta R, et al. Prevalence of and risk factors for chronic kidney disease of unknown aetiology in India: secondary data analysis of three population-based cross-sectional studies. *BMJ Open.* 2019;9(3):e023353.
S25. Singh AK, Farag YM, Mittal BV, Subramanian KK, Reddy SR, Acharya VN, et al. Epidemiology and risk factors of chronic kidney disease in India - results from the SEEK (Screening and Early Evaluation of Kidney Disease) study. *BMC Nephrol.* 2013;14:114.
S26. Trivedi H, Vanikar A, Patel H, Kanodia K, Kute V, Nigam L, et al. High prevalence of chronic kidney disease in a semi-urban population of Western India. *Clin Kidney J.* 2016;9(3):438-43.
S27. Bragg-Gresham J, Thakur JS, Jeet G, Jain S, Pal A, Prasad R, et al. Population-based comparison of chronic kidney disease prevalence and risk factors among adults living in the Punjab, Northern India and the USA (2013-2015). *BMJ Open.* 2020;10(12):e040444.
S28. Srivastava N, Singh RG, Kumar A, Singh S. Insulin resistance in predialytic, nondiabetic, chronic kidney disease patients: A hospital-based study in Eastern Uttar Pradesh, India. *Saudi J Kidney Dis Transpl.* 2017;28(1):36-43.

Supplementary Table S1: Demographics, medical history, and clinical characteristics of participants in the diagnostic assessment

	**Total population**	**Abnormal UACR**
	N=44037	n=15334
**Age (years)**		
Mean [15]	53.5 (13.1)	56.9 (12.7)
Median (range)	54.0 (18.0 - 101.0)	57.0 (18.0 - 101.0)
25th percentile, 75th percentile	45.0, 62.0	48.0, 65.0
Missing	445	106
**Gender**		
Male	25049 (57)	9199 (60)
Female	18513 (42)	6027 (39)
Missing	475	108
**Medical history**		
Diabetes only	13673 (31)	3686 (27)
Hypertension only	6271 (14)	1427 (22.8)
Both diabetes and hypertension	18668 (42)	9353 (50.1)

Data are n (%)
SD, Standard Deviation; UACR, urine albumin-to-creatinine ratio

Supplementary Table S2: Early diagnosis of patients with CKD based on abnormal UACR

Patients with high-risk	Total population (N=44037)	
	Patients with Normal UACR, n (%)	Patients with Abnormal UACR, n (%)
Diabetes only	9657 (21.93)	3686 (8.37)
Hypertension only	4728 (10.74)	1427 (3.24)
Hypertension and Diabetes	9021 (20.49)	9353 (21.24)

Supplementary Figures:

Supplementary Figure S1: Cross-classification of GFR and albuminuria as per KDIGO guidelines

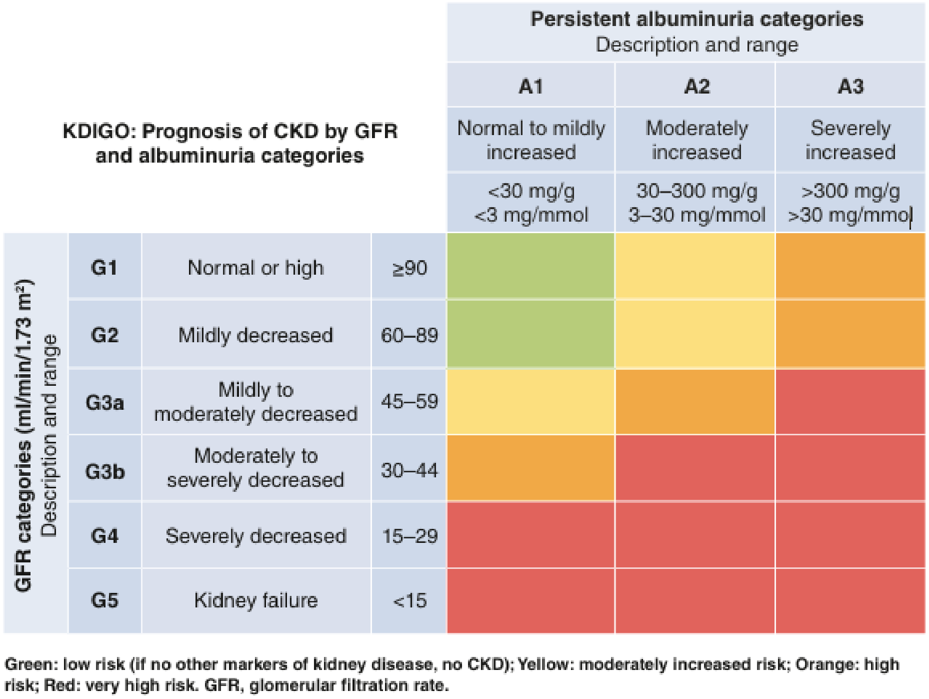

KDIGO: Prognosis of CKD by GFR and albuminuria categories				Persistent albuminuria categories<br>Description and range		
				A1	A2	A3
				Normal to mildly increased	Moderately increased	Severely increased
				<30 mg/g<br><3 mg/mmol	30–300 mg/g<br>3–30 mg/mmol	>300 mg/g<br>>30 mg/mmol
GFR categories (ml/min/1.73 m²)<br>Description and range	G1	Normal or high	≥90			
	G2	Mildly decreased	60–89			
	G3a	Mildly to moderately decreased	45–59			
	G3b	Moderately to severely decreased	30–44			
	G4	Severely decreased	15–29			
	G5	Kidney failure	<15			

Green: low risk (if no other markers of kidney disease, no CKD); Yellow: moderately increased risk; Orange: high risk; Red: very high risk. GFR, glomerular filtration rate.

CKD, chronic kidney disease; GFR, glomerular filtration rate; KDIGO, Kidney Disease Improving Global Outcomes.

Supplementary Figure S2: Project SEARCH: Participating states

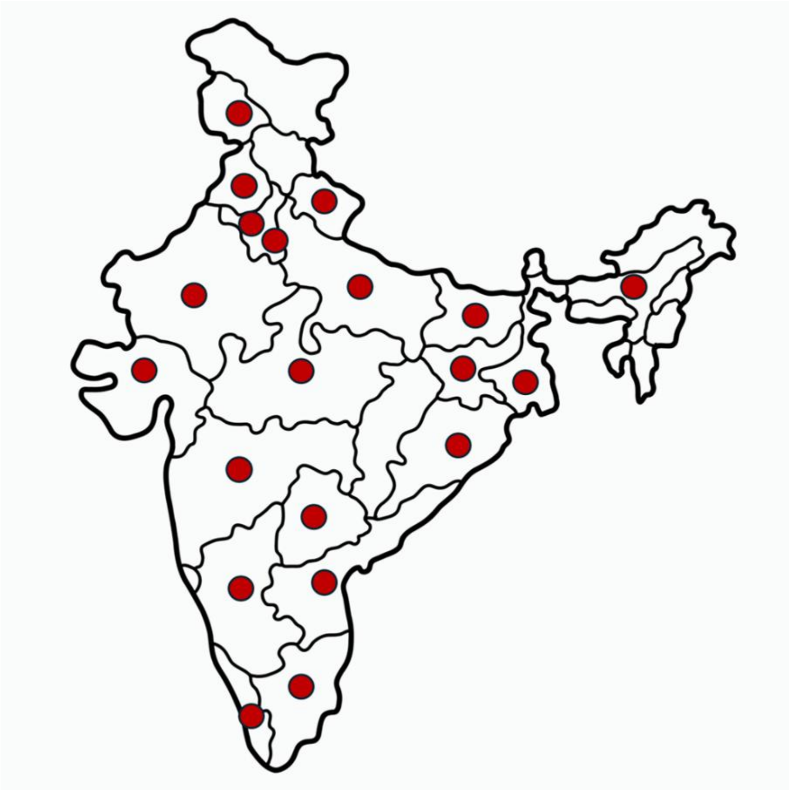

Participating states: Assam, Andhra Pradesh, Bihar, Gujrat, Haryana, Jammu and Kashmir, Jharkhand, Karnataka, Kerala, Madhya Pradesh, Maharashtra, New Delhi, Odisha, Punjab, Rajasthan, Tamil Nadu, Telangana, Uttar Pradesh, Uttarakhand, West Bengal

Supplementary Figure S3: Colour chart on the dipstick bottle to estimate UACR

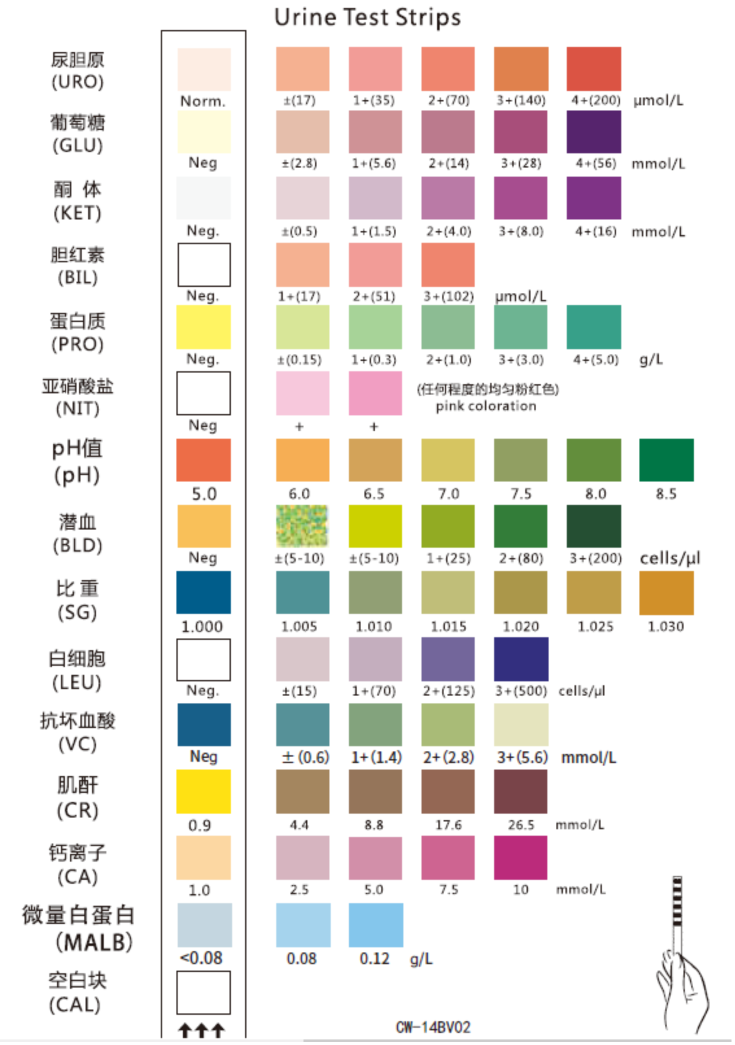

BIL, bilirubin; BLD, blood; CA, calcium; CR, creatinine; GLU, glucose; KET, ketones; LEU, Leukocytes; MALB, microalbumin; NIT, nitrites; PRO, protein; SG, specific gravity; UACR, urine albumin-creatine ratio; URO, urobilirubin; VC, ascorbic acid.

Supplementary Figure S4: Patient record form

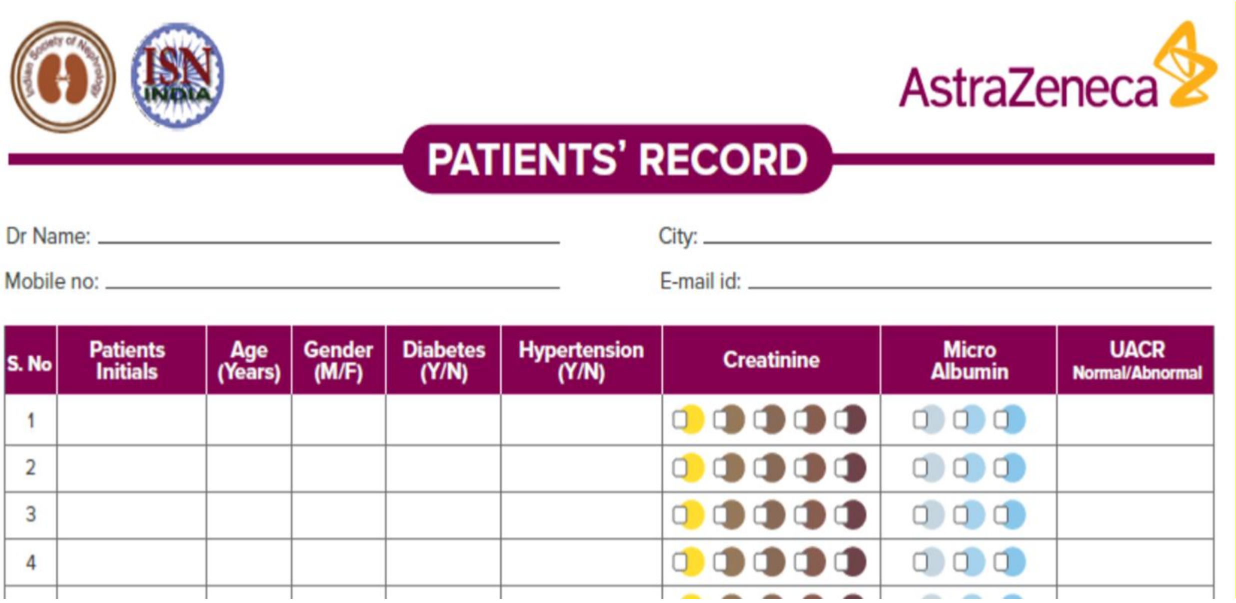

AstraZeneca

PATIENTS' RECORD

Dr Name: ____________________ City: ____________________

Mobile no: ____________________ E-mail id: ____________________

S. No	Patients Initials	Age (Years)	Gender (M/F)	Diabetes (Y/N)	Hypertension (Y/N)	Creatinine	Micro Albumin	UACR Normal/Abnormal
1								
2								
3								
4								

STROBE Statement—Checklist of items that should be included in reports of ***cross-sectional studies***

	Item No	Recommendation	Page No
**Title and abstract**	1	(*a*) Indicate the study's design with a commonly used term in the title or the abstract	1
		(*b*) Provide in the abstract an informative and balanced summary of what was done and what was found	2
**Introduction**			
Background/rationale	2	Explain the scientific background and rationale for the investigation being reported	5-6
Objectives	3	State specific objectives, including any prespecified hypotheses	6
**Methods**			
Study design	4	Present key elements of the study design early in the paper	7 & supplementary page 1
Setting	5	Describe the setting, locations, and relevant dates, including periods of recruitment, exposure, follow-up, and data collection	7 & supplementary page 1
Participants	6	(*a*) Give the eligibility criteria, and the sources and methods of selection of participants	Supplementary page 1
Variables	7	Clearly define all outcomes, exposures, predictors, potential confounders, and effect modifiers. Give diagnostic criteria, if applicable	7 & supplementary page 2
Data sources/ measurement	8*	For each variable of interest, give sources of data and details of methods of assessment (measurement). Describe comparability of assessment methods if there is more than one group	7, supplementary page 2 & 3
Bias	9	Describe any efforts to address potential sources of bias	NA
Study size	10	Explain how the study size was arrived at	Supplementary page 3
Quantitative variables	11	Explain how quantitative variables were handled in the analyses. If applicable, describe which groupings were chosen and why	Supplementary page 3
Statistical methods	12	(*a*) Describe all statistical methods, including those used to control for confounding	Supplementary page 3
		(*b*) Describe any methods used to examine subgroups and interactions	Supplementary page 3
		(*c*) Explain how missing data were addressed	

		(*d*) If applicable, describe analytical methods taking account of sampling strategy	NA
		(*e*) Describe any sensitivity analyses	NA
**Results**			
Participants	13*	(a) Report numbers of individuals at each stage of study—eg numbers potentially eligible, examined for eligibility, confirmed eligible, included in the study, completing follow-up, and analysed	7
		(b) Give reasons for non-participation at each stage	NA
		(c) Consider use of a flow diagram	NA
Descriptive data	14*	(a) Give characteristics of study participants (eg demographic, clinical, social) and information on exposures and potential confounders	7
		(b) Indicate number of participants with missing data for each variable of interest	NA
Outcome data	15*	Report numbers of outcome events or summary measures	7
Main results	16	(*a*) Give unadjusted estimates and, if applicable, confounder-adjusted estimates and their precision (eg, 95% confidence interval). Make clear which confounders were adjusted for and why they were included	7
		(*b*) Report category boundaries when continuous variables were categorized	7
		(*c*) If relevant, consider translating estimates of relative risk into absolute risk for a meaningful time period	NA
Other analyses	17	Report other analyses done—eg analyses of subgroups and interactions, and sensitivity analyses	NA
**Discussion**			
Key results	18	Summarise key results with reference to study objectives	8-10
Limitations	19	Discuss limitations of the study, taking into account sources of potential bias or imprecision. Discuss both direction and magnitude of any potential bias	10
Interpretation	20	Give a cautious overall interpretation of results considering objectives, limitations, multiplicity of analyses, results from similar studies, and other relevant evidence	8-11
Generalisability	21	Discuss the generalisability (external validity) of the study results	11
**Other information**			

Funding	22	Give the source of funding and the role of the funders for the present study and, if applicable, for the original study on which the present article is based	12

*Give information separately for exposed and unexposed groups.

Note: An Explanation and Elaboration article discusses each checklist item and gives methodological background and published examples of transparent reporting. The STROBE checklist is best used in conjunction with this article (freely available on the Web sites of PLoS Medicine at http://www.plosmedicine.org/, Annals of Internal Medicine at http://www.annals.org/, and Epidemiology at http://www.epidem.com/). Information on the STROBE Initiative is available at www.strobe-statement.org.
